# Combination of fruquintinib with venetoclax for the treatment of colorectal cancer

**DOI:** 10.32604/or.2024.050047

**Published:** 2024-12-20

**Authors:** WEI ZHANG, WEICHENG WANG, RUI WANG, XIAO HAN, LIJUN ZHU, WENJIE GUO, YANHONG GU

**Affiliations:** 1 Department of Oncology, The First Affiliated Hospital of Nanjing Medical University, Nanjing, 210029, China; 2 State Key Laboratory of Pharmaceutical Biotechnology, School of Life Sciences, Nanjing University, Nanjing, 210023, China; 3 Department of Oncology, The Affiliated Taizhou People’s Hospital of Nanjing Medical University, Taizhou, 225300, China

**Keywords:** Colorectal cancer (CRC), B-cell lymphoma protein 2 (BCL2), Venetoclax, Vascular endothelial growth factor receptor (VEGFR), Fruquintinib, Anti-angiogenesis, Immunotherapy

## Abstract

**Background:**

As a novel blocker of vascular endothelial growth factor receptor (VEGFR), fruquintinib has been approved for treating colorectal cancer (CRC). However, its dosage and therapeutic efficacy are limited by its widespread adverse reactions. Venetoclax, recognized as the initial inhibitor of B-cell lymphoma protein 2 (BCL2), has shown potential in boosting the effectiveness of immunotherapy against CRC. This study investigated the efficacy and mechanisms of fruquintinib combined with venetoclax in treating CRC.

**Methods and Materials:**

We developed a colon cancer mouse model with the CT26 colon cell line to demonstrate fruquintinib and venetoclax’s efficacy against tumors. Then we employed various techniques to evaluate different aspects of the experimental outcomes. Immunohistochemistry was used to detect cell proliferation and angiogenesis in tumor tissues. Western blot analysis was utilized to examine the occurrence of cell apoptosis, and flow cytometry to quantitate immune cells within the tumor tissues. Moreover, immunofluorescence was employed to measure cytokine levels.

**Results:**

The strongest inhibition on tumor growth was achieved by the combination of fruquintinib with venetoclax, as opposed to individual drug use. Venetoclax was found to amplify the impact of fruquintinib, leading to decreased cancer cell proliferation, increased cancer cell apoptosis, lowered angiogenesis, better vascular structure normalization, and improved immune cell infiltration.

**Conclusion:**

Our findings indicate that the addition of venetoclax enhances the impact of fruquintinib on vascular normalization and modulation of the tumor immune microenvironment. Our study presents the justification for utilizing the fruquintinib and venetoclax combination in treating CRC. Venetoclax holds promise in being assimilated into anticancer medications for CRC.

## Introduction

Colorectal cancer (CRC) ranks as the third most common and the second most lethal form of malignant tumors, presenting a significant global health challenge [1]. At the time of diagnosis, about a quarter of CRC patients are already battling metastases, and nearly half will ultimately develop metastatic disease. The prognosis for patients with metastatic colorectal cancer (mCRC) is grim, with a five-year survival rate ranging from a mere 5% to 15% [2]. Systemic therapies are recommended for advanced-stage CRC, typically involving first-line and second-line drugs, such as fluorouracil, oxaliplatin, irinotecan, bevacizumab, and cetuximab [3]. Despite these options, the effectiveness of such treatments is significantly hampered by the emergence of drug resistance and the occurrence of adverse reactions, often leading to the recurrence and progression of metastasis.

In recent years, the introduction of immune checkpoint inhibitors targeting the programmed death receptor 1 (PD-1)/programmed cell death ligand 1 (PD-L1) axis and cytotoxic T lymphocyte-associated protein 4 (CTLA-4) has broadened the spectrum of therapeutic strategies for CRC, notably for its metastatic variant (mCRC). Anti-PD-1 shows its superiority in dealing with tumors displaying the microsatellite instability-high (MSI-H) phenotype, a characteristic found in only 4–6% of cases of advanced colorectal cancer. Patients with microsatellite stable (MSS) mCRC have not shown favorable responses to monotherapy with these immunotherapies in previous clinical trials [4–6]. Moreover, after undergoing more than three-line therapies, many patients experience a marked deterioration in their physical well-being, rendering them unable to continue with prolonged treatment regimens. Consequently, there is a pressing need to develop later-line treatment options for mCRC that not only minimize adverse effects but also enhance therapeutic efficacy.

Currently, the standard third-line drugs recommended for mCRC include regorafenib, fruquintinib, and TAS-102 [7–9]. Fruquinitinib, an oral tyrosine kinase inhibitor (TKI), is highly selective in inhibiting VEGFR 1, 2, and 3. Fruquinitinib can effectively suppress VEGFR phosphorylation, which in turn blocks tumor angiogenesis and tumor growth. Fruquintinib has received approval from the United States Food & Drug Administration and is endorsed by the National Comprehensive Cancer Network guidelines for the management of mCRC in adults who have previously received a fluoropyrimidine, oxaliplatin, and irinotecan-based chemotherapy, an anti-VEGF therapy, and, if having a RAS wild type and being medically suitable, an anti-EGFR therapy. Many clinical studies have shown that fruquintinib monotherapy can effectively prolong the survival time of patients with advanced CRC [7,10]. Fruquintinib also enhances the antitumor immune response against PD-1 in CRC [11–13].

Programmed cell death, a complex cellular dynamics, is involved in antitumor immune responses. The family of B-cell lymphoma protein 2 (BCL2) regulates apoptotic signaling pathways enriched in immune cell development, homeostasis, and responses [14]. Apoptosis, a key mechanism within programmed cell death, is vital for maintaining tissue development. Any disruption in cell apoptosis may trigger the progression of tumors [15]. Members of the *BCL2* gene family, owing to their close tie with cell apoptosis, have slipped into the research hotspot. Some BCL2 members can effectively inhibit programmed apoptosis in cells and then induce malignant tumors [16]. BCL2, cloned from the breakpoint of the human B-cell lymphoma translocation, is the first identified family member [17]. Numerous studies have indicated that BCL2 is differentially expressed in human tumors, exerting a direct or indirect impact on the proliferation, migration, invasion, and development of tumor cells at different stages of apoptosis [18]. Studies have shown that BCL2 expression is linked to cancer progression, specifically liver metastases of CRC [19].

Venetoclax, also known by its names ABT-199, Venclexta, and Venclyxto, is an orally administered, highly selective BCL2 inhibitor capable of triggering tumor cell apoptosis. The US Food and Drug Administration has endorsed venetoclax for treating chronic lymphocytic leukemia and acute myeloid leukemia [20]. Venetoclax can selectively target to inhibit BCL2, restore the apoptotic process, replace pro-apoptotic proteins, and impart cytotoxicity on BCL2-overexpressed tumor cells [21,22], ultimately contributing to the killing of tumor cells [23]. Encouraging efficacy has been achieved by therapies combining multiple BCL2 family inhibitors simultaneously, or BCL2 family inhibitors with standard anticancer drugs. Currently, venetoclax is most preferred for blood system diseases such as leukemia; however, relevant clinical trials for solid tumors, such as small cell lung cancer, colorectal cancer, and breast cancer, are also underway.

In our study, a syngeneic CRC model was developed to evaluate the effectiveness of combining a BCL2 inhibitor with an antiangiogenic drug. The therapeutic mechanism related to immunosuppressive tumor microenvironment was also elucidated. This combination might serve as a therapeutic strategy for CRC.

## Materials and Methods

### Reagents

Fruquintinib used in this study was supplied generously by Hutchison MediPharma Limited (Shanghai, China). Venetoclax was purchased from AbbVie Inc. (North Chicago, Illinois, USA). The TUNEL Cell Apoptosis Detection Kit (G1501-50) was obtained from Servicebio Technology (Wuhan, Hubei, China). CD45-BV421 (103134), CD4-PE/Dazzle 594 (100456), CD8-FITC (100706), and IFN-γ-APC (505809) used for flow cytometry were obtained from Biolegend (San Diego, California, USA). In the immunohistochemistry and immunofluorescence, antibodies were used as follows: anti-PCNA (sc-56; Santa Cruz, California, USA), anti-CD31 (28083-1-AP; Proteintech, Rosemont, Chicago, USA), anti-α-SMA (55135-1-AP; Proteintech), anti-Ki67 (343569; Abmart, Shanghai, China), anti-granzyme B (10345; Sino Biological, Beijing, China), and anti-IFN-γ (105995; Sino Biological). For immunofluorescence, the secondary antibody used was CoraLite488-conjugated goat anti-rabbit IgG (SA00013-2) from Proteintech. Immunohistochemistry (GK500705) was operated on the GTVision kit (GeneTech Company, Shanghai, China). The monoclonal antibodies (mAbs) for western blot analysis included Cleaved Caspase 3 (AC030-1; Beyotime, Shanghai, China), β-actin (T0022; Affinity Biosciences, Changzhou, Jiangsu, China), anti-mouse IgG (H+L) (5220-0341; Milford, Massachusetts, USA) and anti-rabbit IgG (H+L) (5220-0336; Milford). Other reagents for our experiments were purchased from Beyotime.

### Cell culture

The CT26 cells were sourced from the Shanghai Institute of Cell Biology and cultured in RPMI 1640 medium (11875-093, Gibco) containing 10% heat-inactivated fetal bovine serum (C04001-500, Vivacell), penicillin (100 U/mL), and streptomycin (C0222, Beyotime, China) (100 mg/mL) at 37°C in a 5% CO_2_ and humidified environment. Cell quality analysis for the CT26 cells was performed by a third-party company (Jiangsu KeyGEN BioTECH Corp., Ltd). For mycoplasma testing, the Certificate of Analysis for CT26 cells indicated that the cells did not have mycoplasma infection.

### Animal model establishment

24 Seven-week-old BALB/c mice were acquired from SPF BIOtechnology Co. LTD in Suzhou, Jiangsu, China, kept in plastic cages, and given free access to pellet food and water at 21°C ± 2°C in a 12-h light/dark cycle. The animal experiment protocol was approved by the Animal Experiment Ethical Review Committee of Nanjing Medical University, China (Approval number: IACUC-2210010). All animal experiments were conducted according to the guidelines of the Animal Experimentation Center and under the review and supervision of Nanjing Medical University (Approval number: SYXK2023-0029). Minimized animals were used and their suffering was maximally controlled. Tumors were induced by inoculation of CT26 cells (1 × 10^6^) into the right flank of BALB/c mice for three days. After the tumor reached 50 mm^3^ in volume, the mice were split into four groups with 6 mice in each group. Fruquintinib dosed at 1.5 mg/kg was dissolved in 0.9% sodium carboxymethyl cellulose and given once daily, while venetoclax dosed at 50 mg/kg was dissolved in 0.9% sodium carboxymethyl cellulose and also given once daily. Both drugs, 100 μL each, were simultaneously used in the combination group. The vehicle group received 100 μL of 0.9% sodium carboxymethyl cellulose only. Tumor volume was calculated by a ∗ b ∗ b ∗ 0.5, where “a” represents the maximal orthogonal length and “b” represents the maximal orthogonal width, which was measured every two days using a vernier caliper. After delivery of drugs for 13 days. The mice were weighed and euthanized on the 13th day, and their tumors were removed and weighed.

### Preparation of tumor-infiltrating cells and intracellular staining

Levels of tumor-infiltrating cells in each group were measured as previously reported [24]. The mouse tumor tissues were harvested, followed by mechanical and enzymatical dissociation using a dissociation kit (130-096-730, Miltenyi Biotec, Germany). The suspensions were filtered through a 70-μm cell strainer. Single-cell suspensions were labeled with different surface monoclonal antibodies, such as anti-CD45 conjugated with BV421, anti-CD4 conjugated with PE/Dazzle 594, and anti-CD8 conjugated with FITC. To analyze IFN-γ, the cells were pre-cultured in 24-well, flat-bottom plates with a stimulation mixture under optimized conditions. Then the cells were fixed and permeabilized. After permeabilization, the cells were labeled with intracellular markers of APC-IFN-γ. At the end of staining, cells were washed once using PBS and then flow cytometry was performed.

### Western blot analysis

Proteins were extracted from whole tumor tissues using RIPA lysis (P0013B, Beyotime, Shanghai, China) buffer mixed with protease inhibitor cocktail and kept on ice. Proteins in an equal amount determinated by a BCA protein assay kit (23228, Thermo, USA) were isolated using SDS-PAGE, and then shifted to polyvinylidene difluoride membranes, which were then blocked with 3% BSA (B0012-100, SunShine Bio, Nanjing, China), incubated with primary antibodies (1:1000) at 4°C overnight, and then with secondary antibodies (1:10000, RT). Later on, immunoreactive bands were identified by using an ECL kit (180-506, Tanon, Shanghai, China), following the guidelines provided by the manufacturer.

### Hematoxylin and eosin (H&E) staining, immunohistochemistry (IHC), immunofluorescence (IF) and TUNEL assay

After deparaffinization, rehydration, and rinsing with 1% PBS-Tween 20, the sections were subjected to IHC, in which hydrogen peroxide (2%) was introduced to block endogenous peroxidases, followed by blocking with goat serum (C0265, Beyotime, Shanghai, China) (3%), as well as incubation with specific primary Abs (1:200) at 4°C overnight. After another round of incubation with streptavidin–HRP for 40 min, the sections were first stained with diaminobenzidine substrate and then counter-stained with hematoxylin. Afterward, immunofluorescence and TUNEL were performed by staining the sections with fluorescence-labeled TUNEL-FITC (1:100), followed by counter-staining with DAPI (C1002, Beyotime, Shanghai, China) for 5 min. Imaging was done using light microscopy and fluorescence microscopy (BX51, Olympus, Tokyo, Japan).

### Statistical analysis

All statistical analyses were conducted using GraphPad Prism version 9.0 (La Jolla, CA, USA). Results were expressed as mean±SEM, with significance set at *p* < 0.05. Two-tailed Student’s *t*-test was used for analyzing continuous variables. Wilcoxon test was employed for comparing two groups, while the Kruskal-Wallis test was utilized for evaluating differences among three groups. Non-repeated measures ANOVA followed by Scheffe test was applied for cases with more than three groups.

## Results

### The growth of transplanted CRC in mice was significantly inhibited by combining venetoclax with fruquintinib

**Figure 1:**
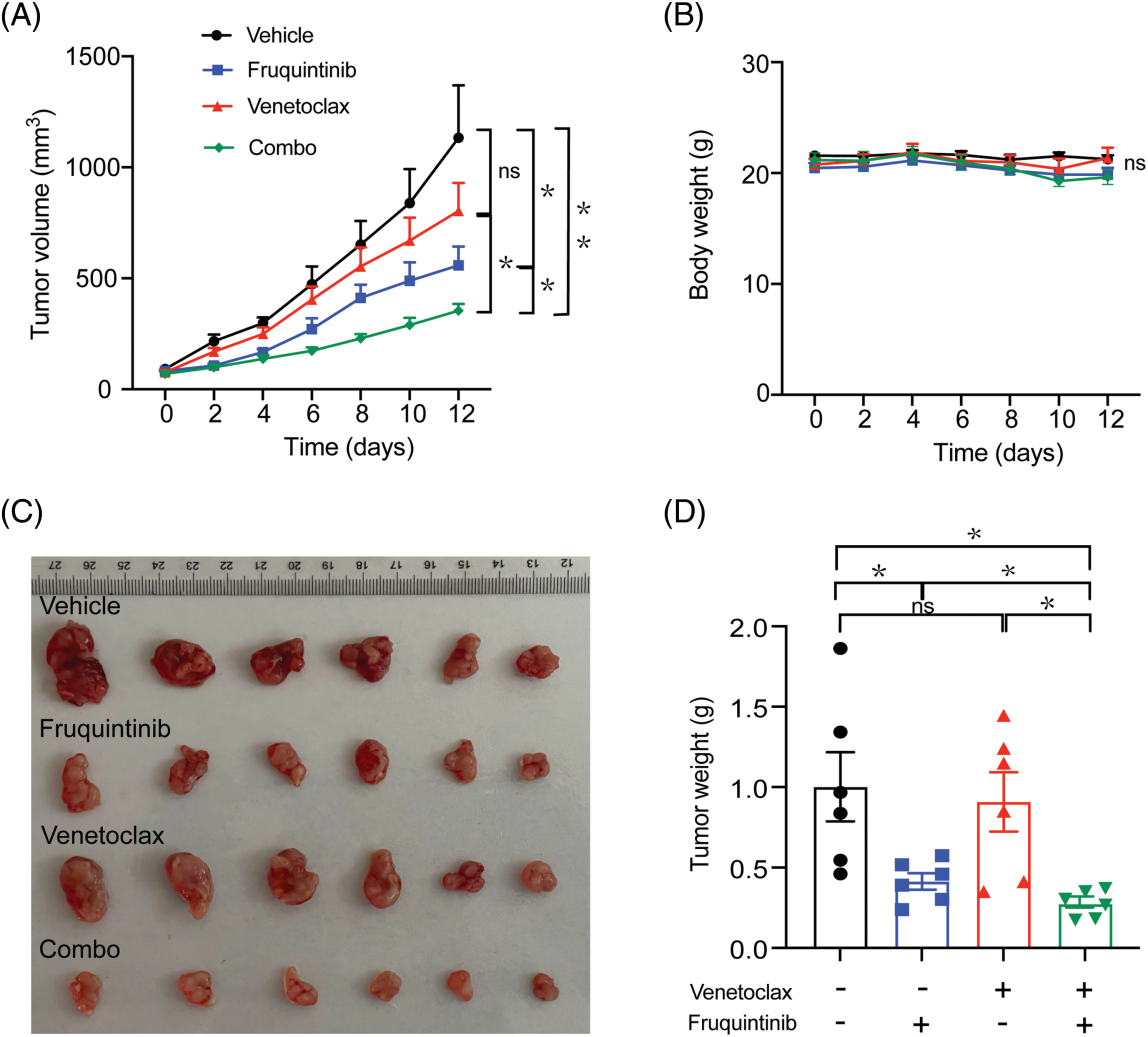
The growth of transplanted tumors in mice was significantly inhibited by the combination of fruquintinib with venetoclax. The weight of the bodies and volumes of tumors were measured every other day (A and B). Following sacrifice, solid tumors from the mouse were isolated, photographed, and weighed (C and D). Results from six mice per group were indicated as mean ± SEM. *p < 0.05, **p < 0.01 compared to indicated values. ns, not significant.

A xenograft murine model was established by transplanting CT26 mouse CRC cells subcutaneously. Venetoclax was delivered at a dose of 50 mg/kg, and fruquintinib at a dose of 1.5 mg/kg, both orally once every day. Mice treated with the combination of venetoclax with fruquintinib exhibited slower tumor growth and a lower tumor weight, compared to the control group (Figs. 1A, 1C, and 1D). Notably, fruquintinib reduced tumor weight by 50.7%, venetoclax by 29.1%, and their combination by 68.76%. During the administration period, there was no significant change in body weight observed in all the combination groups (Fig. 1B). This suggested that the combination of venetoclax and fruquintinib had little to no side effects in the mice.

### A combination of venetoclax with fruquintinib inhibited the proliferation of CRC cells in vivo

**Figure 2:**
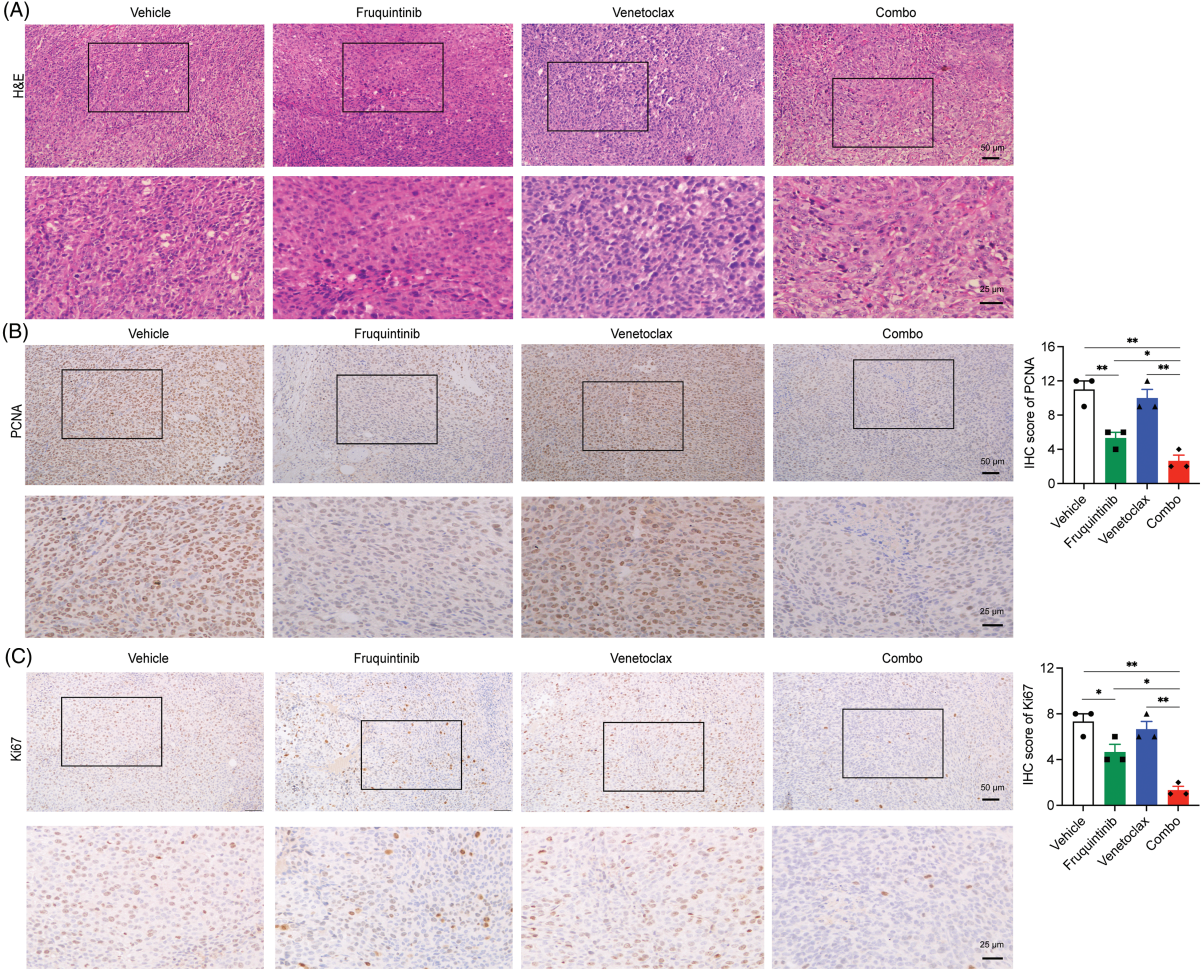
Combination of venetoclax with fruquintinib inhibited the proliferation of tumor cells *in vivo*. (A) Tumor tissues were examined using H&E staining on paraffin sections. (B) The assessment of PCNA-positive cells was conducted via IHC with ImageJ in three random fields. (C) We analyzed the quantification of Ki67-positive cells through immunohistochemistry (IHC) with ImageJ in three random fields. The data of three mice per group were indicated as the mean ± SEM. *p < 0.05, **p < 0.01, *vs*. as indicated.

H&E staining revealed that the combination distorted tumor cell morphology significantly, including nuclear shrinkage, sparse arrangement, and cell fragmentation (Fig. 2A). Through further immunohistochemical analysis, the protein levels of PCNA and Ki67 in the tumor tissues were lower in the combination group than in the fruquintinib single-agent group (Figs. 2B and 2C). These findings confirmed that the addition of venetoclax enhanced the tumor-inhibitory effect of fruquintinib.

### Venetoclax enhanced the effects of fruquininib on inhibiting tumor neovascularization and inducing apoptosis of cancer cells in vivo

**Figure 3:**
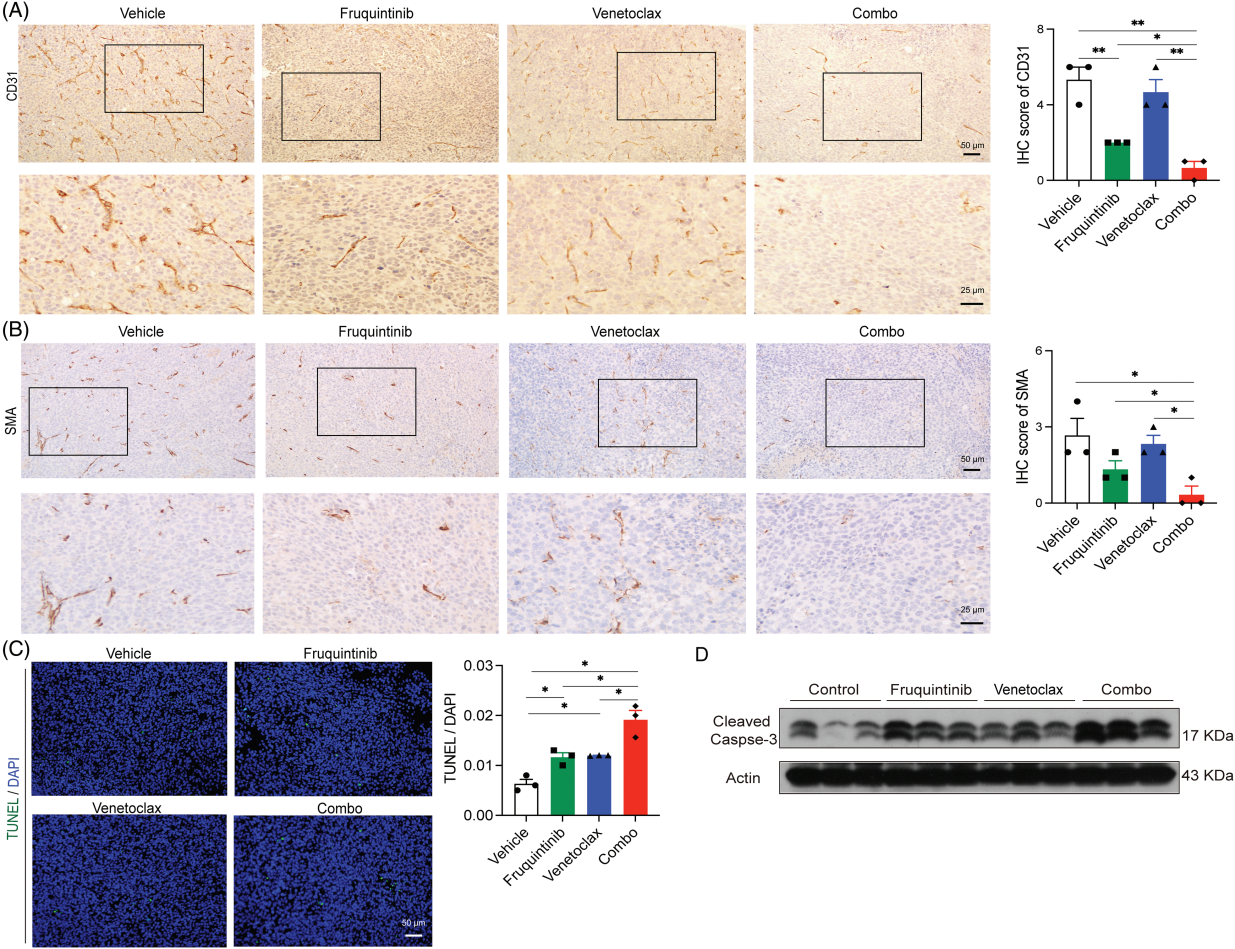
The addition of venetoclax promoted the antiangiogenic effect of fuquintinib, and the combination of both made the effect of promoting apoptosis superimposed. (A) IHC-examined expression of PECAM-1 (CD31) in CT26 tumor tissues was examined by IHC. (B) IHC-examined expression of α-SMA in CT26 tumor tissues was examined by IHC. (C) TUNEL staining of CT26 tumor tissues. (D) Western blot analysis of cleaved-Caspase 3. *p < 0.05.

Next, we investigated whether venetoclax can enhance the effect of VEGFR-targeting fruquintinib. IHC was performed to detect the PECAM-1 (CD31) and α-SMA expression. PECAM-1 (CD31) is a tumor neovascularization marker and α-SMA is a vascular smooth muscle cell marker in mouse colon cancer tissues. The results showed that although fruquintinib itself has a good anti-angiogenic effect, the addition of venetoclax further reduced the level of PECAM-1 (CD31) and reduced the expression of α-SMA (Figs. 3A and 3B). Furthermore, TUNEL staining and Western blot analysis of cleaved Caspase 3 confirmed that the combination inflicted tumor cells with extensive apoptosis (Figs. 3C and 3D). These findings suggested that the addition of venetoclax enhanced the anti-angiogenic effects of fruquintinib and induced cancer cell apoptosis in mouse CRC tissues.

### Venetoclax promoted the anti-tumor immune function of fruquininib

**Figure 4:**
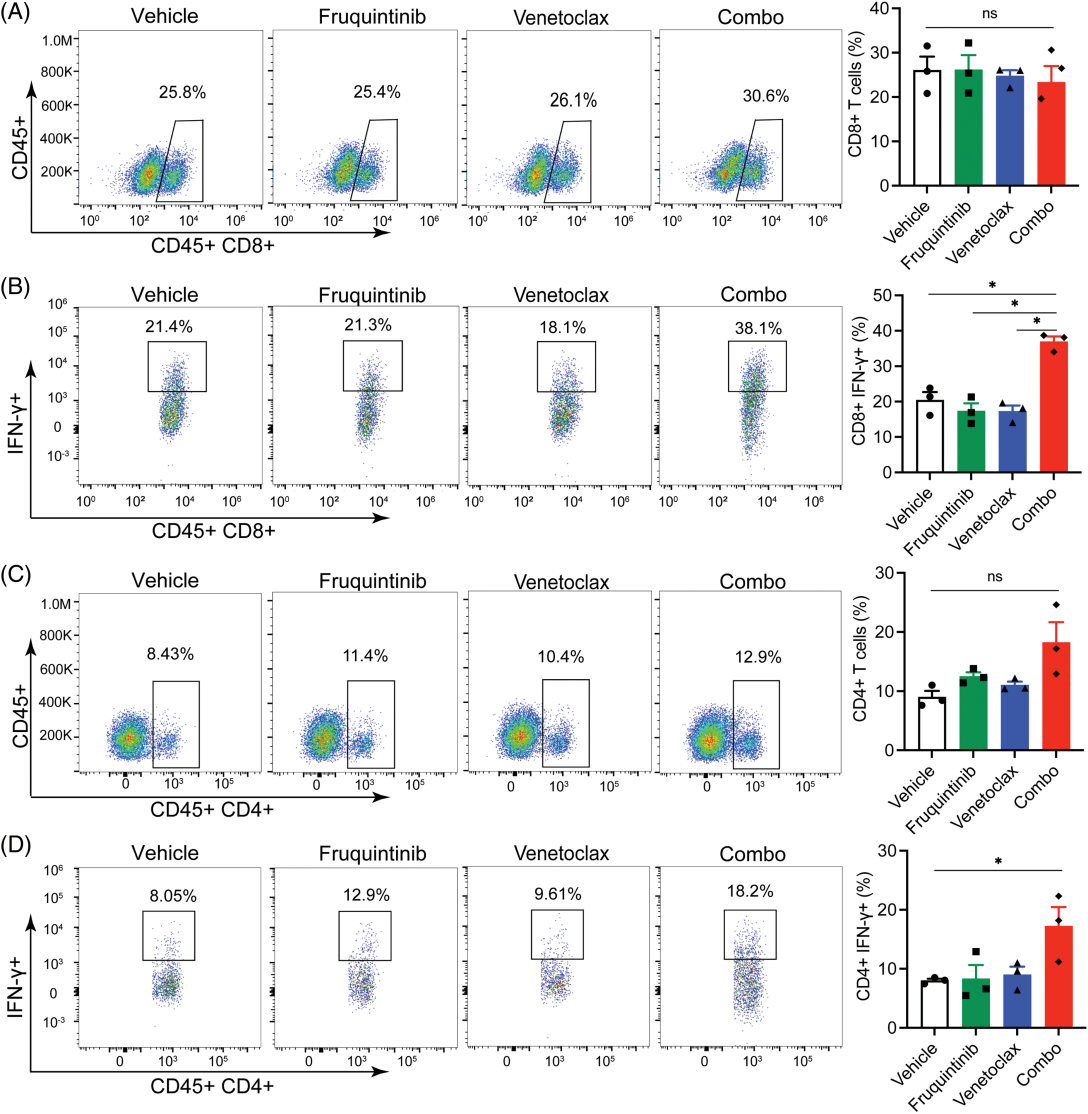
Combination of venetoclax with fruquintinib substantially activated CD4^+^ and CD8^+^ cells in tumor tissues. Percentages of CD45^+^CD8^+^ (A) and CD45^+^CD4^+^ T cells (C) in a viable lymphocyte gate. Counts of CD45^+^CD8^+^IFN-γ^+^ (B) and CD45^+^CD4^+^IFN-γ^+^ T cells (D) in a viable lymphocyte gate. The data of three mice per group were indicated as the mean ± SEM. *p < 0.05, *vs*. as indicated. ns, not significant.

**Figure 5:**
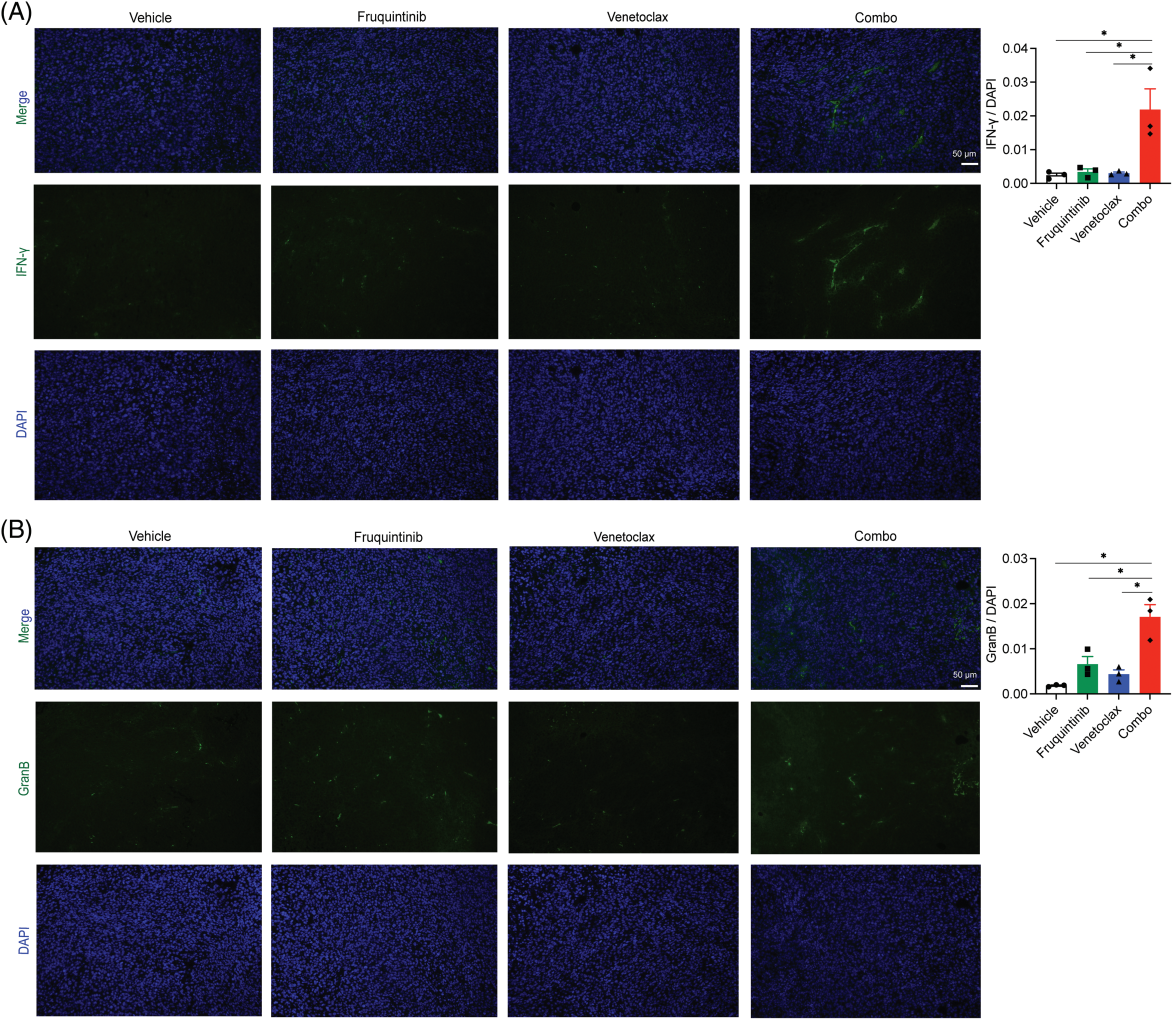
Venetoclax increased the expression of IFN-γ and Granzyme B caused by fruquintinib. Representative images of IFN-γ (A) and Granzyme B (B) (green) immunostaining and DAPI nuclear staining (blue) in tumor tissues. The data were expressed as the mean ± SEM of three mice per group. *p < 0.05.

We further profiled the abundance and functions of immune cells within tumor tissues. No significant changes were witnessed in the levels of CD45^+^CD8^+^ T cells and CD45^+^CD4^+^ T cells (Figs. 4A and 4C), but those of CD45^+^CD8^+^IFN-γ^+^ T cells and CD45^+^CD4^+^IFN-γ^+^ T cells increased obviously (Figs. 4B and 4D), indicating activation of these immune cells in the tumor microenvironment (TME) upon the induction from the combination of venetoclax and fruquintinib. Furthermore, the abundance of markers of cytotoxic lymphocytes, such as IFN-γ and granzyme B, rose in the fruquintinib-treated mice and peaked after administration of both drugs (Figs. 5A and 5B). The findings suggested that the co-treatment of venetoclax and fruquintinib might provoke immune activation in the TME to realize its antineoplastic effects.

## Discussion

MSS-CRC is the most common type of tumor in both nonmetastatic and metastatic situations. Thus far, single use of anti-PD-1/PD-L1 has never demonstrated effectiveness in treating these tumors. Fruquintinib, a blocker of VEGFR, commonly utilized in the treatment of mCRC, restores the vessels of tumors in a dependent manner of dose and time, which is a crucial factor in the impact of combining fruquintinib with cytotoxic medications. Several international and multicentre clinical studies showed that treatment with fruquinib demonstrated a notable and important improvement in survival outcomes among individuals diagnosed with advanced colorectal cancer who did not respond to previous therapies [7,10,25]. Studies have demonstrated that the concomitant administration of fruquintinib and an anti-PD-1 antibody powerfully inhibits CRC growth [11,26]. This combination works by modifying the tumor immune microenvironment, ultimately supporting its use in the treatment of advanced CRC. However, fruquintinib presents a variety of adverse drug reactions, including hypertension, hand and foot skin reaction (HFSR), diarrhea, and thrombocytopenia, which are frequently observed in clinical practice [7,25]. Although these adverse reactions are relatively controllable, it is not easy to improve the efficacy of fruquintinib by increasing the dose.

Apoptosis is a meticulously regulated fundamental cellular process that takes place in both physiological and pathological situations, aiding in the removal of abnormal cells to uphold homeostasis. Apoptosis is also a crucial process in immune system development and self-regulation. Traditionally viewed as an active biological mechanism, apoptosis has been a focal point in cancer research for prevention and treatment [27,28]. Oncoprotein BCL2 is essential for tumor development and the activation of the immune system [14,29,30]. Aside from hematopoietic malignancies, the *BCL2* gene exhibits activity in various types of solid tumors, including but not limited to breast cancer, lung cancer, pancreatic cancer, and sarcoma. Therefore, BCL2 inhibitors may be manipulated to treat solid tumors. In recent years, BCL2 inhibitors have made great progress in the treatment of hematologic malignancies. However, the effectiveness of monotherapy with BCL2 inhibitors in solid tumors is currently limited, with the majority of studies still in the preclinical stage. Therefore, it is imperative to assess the potential of combination therapy as a promising future direction [31]. Our study indicates that combining the BCL2 inhibitor venetoclax with fruquintinib could enhance immune responses in the TME, potentially inhibiting CRC progression. It was reported that venetoclax could enhance antitumor T-cell responses when used in combination with immune checkpoint inhibitors [32]. Meanwhile, combinations of BCL2 inhibitors with other anticancer drugs have also been reported. For example, a combination of BCL2 inhibitor Navitoclax with a third-generation EGFR inhibitor osimertinib has demonstrated high safety and feasibility in patients with advanced EGFR-mutated lung cancer [33]. In another study, introducing BCL2 inhibitors into chemotherapy achieves favorable effects in patients with esophageal cancer, suggesting their potential to be widely used in solid tumor treatments. *In vitro* experiments have shown that BCL2 inhibitor AT-101 can reduce tumor cell proliferation and enhance the apoptotic effect of docetaxel. Additionally, preclinical and clinical data suggest that AT-101 can repress tumor cell proliferation and promote docetaxel-induced apoptosis, potentially overcoming resistance in gastroesophageal cancers [34]. Studies have shown that increased expression of BCL2 comes with T-cell activation and cellular adhesion [35]. BCL2 is implicated in regulating T-cell homeostasis and effector responses [14]. Mouse model studies show that BCL2 knockout reduces lymphocyte counts, but effector lymphocytes, once survived, can still proliferate upon immune stimulation, indicating BCL2’s partial role in immune responses [36,37]. However, the role of BCL2 in these processes remains controversial. In the mouse model, venetoclax enhances the ability of immune checkpoint inhibitors in fighting against tumors and increases PD-1+ T memory effector cell levels without impairing T cell function or antagonizing anti-PD-1-induced activation. Estrogen receptor (ER)-positive breast cancer is the first solid tumor type to undergo clinical trials with venetoclax. In 2013, venetoclax was found to significantly improve tumor responses to tamoxifen and mitigate specific adverse effects of tamoxifen in xenografts of ER-positive breast cancer [38]. In a study on small cell lung cancer (SCLC) characterized by high invasiveness and a poor prognosis, researchers found that the upregulation of BCL2 expression was common and could be used as a predictor of drug sensitivity [39]. Another study found that the combination of the BET inhibitor ABBV-075 and venetoclax showed a strong synergistic inhibitory effect on SCLC, and this effect was positively correlated with the expression of BCL2 [40].

The TME consists of tumor cells, extracellular matrix, immune cells, cytokines, blood vessels, and lymphatic networks. Tumor cells are a key component of the TME, serving as both the main body of the tumor and the initiator of tumor development. Tumor cells exhibit characteristics such as excessive proliferation and inhibition of apoptosis [41,42]. Excessive proliferation often leads to hypoxia, mitochondrial damage, and the expression of Reactive oxygen species (ROS) and transcription factor 1α (HIF-1α). HIF-1α triggers the production of pro-angiogenic factors like VEGF, promoting new blood vessel formation, as well as transcription activation of TGF-β3 and epidermal growth factor (EGF) to facilitate tumor metastasis to oxygen-rich tissues. Additionally, the migration ability of tumor cells can promote local invasion, lymphatic migration, or blood migration [43]. Metabolically, tumor cells primarily rely on aerobic glycolysis, generating lactic acid and creating an acidic microenvironment that suppresses the immune response, further enhancing tumor metastasis and recurrence. Early in 1998, a study suggested that BCL2 possibly controls the development of tumor angiogenesis with putative mediation by VEGF in lung cancer [44]. A study demonstrated that increased levels of HIF-1α are linked to elevated levels of BCL-xl, a member of the BCL2 family, primarily as a result of its anti-apoptotic characteristics, leading to the development of resistance to chemotherapy and radiation [45].

It has been reported that BCL2 expression in lymphoma cells induces hypoxia-inducible factor-1 alpha (HIF1α)-mediated expression of VEGF, an angiogenic factor that promotes endothelial cell proliferation and migration. There was evidence that BCL2 prevents HIF-1α degradation in a contact-dependent process under non-hypoxic conditions, and this binding promotes dimerization of HIF-1α and HIF-1β, subsequently stimulating through interactions facilitated by the BH4 region, the physical connection between BCL2 and HIF-1α serves to underscore the potential impact of BCL2 mutations on the signaling pathways of HIF-1α and VEGF [46]. The specific mechanism of venetoclax combined with fruquintinib was not thoroughly researched in our study, and the combination of the two drugs is expected to be further explored in clinical practice in the future.

In summary, there is currently no regimen for the combined use of antiangiogenic drugs and BCL2 inhibitors. Our study examined the effects of venetoclax and assessed its combined efficacy with the VEGFR inhibitor fruquintinib in treating CRC [47]. Our findings showed that combining venetoclax with fruquintinib more effectively inhibited tumor growth, reduced tumor cell proliferation, and induced apoptosis than venetoclax alone, while also hindering new blood vessel formation fruquintinib alone. Although the combination therapy did not significantly increase T cell infiltration, it enhanced T lymphocyte activity. VEGFR inhibitors block angiogenesis, and nutrient delivery to tumors, and induce cancer cell death and tumor antigen release. The addition of venetoclax promotes these effects. BCL2 may play a dual role in tumor immunotherapy. On the one hand, it may be an anti-immune factor of the tumor, and on the other hand, it may be a potential target of immunotherapy, which can enhance the effect of immunotherapy by inhibiting its function. Together, our findings and previous studies indicate that combining a BCL2 inhibitor with a VEGFR inhibitor could be a promising strategy for CRC treatment.

## Conclusion

This is the first time that the addition of venetoclax enhances the impact of fruquintinib on vascular normalization and modulation of the tumor immune microenvironment. This study presents the justification for utilizing the fruquintinib and venetoclax combination in treating CRC. Venetoclax may potentially be integrated into anticancer therapies.

## Data Availability

Data are available upon reasonable request.
